# Investigating false start of the main growing season: A case of Uganda in East Africa

**DOI:** 10.1016/j.heliyon.2021.e08428

**Published:** 2021-11-19

**Authors:** Emmanuel Ocen, C.A.J.M. de Bie, Charles Onyutha

**Affiliations:** a ITC, University of Twente, Enschede, the Netherlands; b Department of Civil and Environmental Engineering, Kyambogo University, P.O. Box 1, Kyambogo, Kampala, Uganda

**Keywords:** False start of growing season, Precipitation variability, Agronomic drought

## Abstract

False start of the growing season (Fsos) is a component of the onset variability related to agronomic drought that adversely impact on agricultural production and productivity. In the sub-Saharan Africa (SSA) where agriculture heavily depends on rainfall, the Fsos tends to create confusion among farmers on when to start planting crops thereby affecting seed germination and normal growth after emergence. In this paper, we focus on the Fsos and the occurrence of dry spell especially before the Start of growing Season (SoS). We take advantage of the existing rainfall estimates (CHIRPS) and remotely sensed data for vegetation performance (NDVI) over the period 1999–2017 in combination with local knowledge derived from farmers to map out areas at risk of (i) dry spell at the SoS, and (ii) false timing of SoS or high probability of occurrence of the Fsos. We found that the North Eastern part of Uganda (8.8% of arable area) were at risk of dry spell throughout each year. However, the greater North (58.1% of arable area) was prone to dry spell during the onset of the March–May season. Areas in the South Western (3.7%) region were at risk during the onset of the September–November season. The probability that a location in Uganda experiences an Fsos falls between 0-53%. The findings in this study are vital for planning of predictive adaptation to the impacts of climate variability on agriculture amid struggle aimed at tackling food insecurity challenge in the SSA.

## 1 Introduction

Food production especially in the sub-Saharan region heavily relies on rainfed agriculture; and this will even continue under future climatic conditions (FAO, 2011). Due to climate change, the frequency, intensity, and severity of extreme climate events are likely to increase (IPCC, 2007). Limitation (or absence) of rainfall leads to dry spell (rainfall breaks) thereby affecting the vegetation growing period since the rainfall distribution can be irregular and difficult to predict. Such an erratic (or unpredictable) rainfall can cause heavy economic losses to smallholder farmers, thus, resulting in food insecurity and poor livelihood (Bates et al., 2008; Winkler et al., 2017). In the sub-Saharan Africa (SSA), farmers predominantly rely on rainfed cropping systems and the rainfed crops cover more than 95% of the cultivated land (FAO, 2011; World Bank, 2007). The limitation of irrigated crops to less than 5% of the arable land in the SSA is because of the rampant household poverty (Onyutha, 2018a). Nevertheless, due to climate variability (the impact of which casts a worrying situation in the fight against food insecurity in the SSA), rainfall is increasingly becoming unreliable to meet the crop water requirements (Onyutha, 2018a). The climatic conditions of Uganda have been characterized by extreme weather phenomena, particularly those related to precipitation (Kaggwa et al., 2009). These phenomena which tend to be manifested in terms of increasing frequency and duration of droughts, storms, and floods directly affect agricultural productivity by reducing yields and hence less food to meet the needs of the increasing population. Early or delayed onset of rainfall greatly affects planning and decision of the smallholder farmers regarding when to plant their crops. Also, rainfall distribution and cessation essentially affects growth and yield (Getachew and Teshome, 2018). Therefore, the correct determination of the start of the growing season is important to ensure that there is an adequate soil moisture to support seed emergence after planting and crop growth (Ati et al., 2002; Orlove et al., 2010).

Previous studies on onset of rainy seasons adopted varying approaches depending on the driving forces for a specific region. For instance, Stern (1981) determined an onset of the rainy season by fitting a model to daily rainfall allowing derivation of the probability of a particular event including dry spell. In this way, the start of rains would be the day in which cumulative total of 20mm can be obtained following one or two consecutive days (Stern, 1981). Dodd and Jolliffe (2001) defined the onset as the first period of 5 consecutive days in which atleast 25mm of rain is received and not followed by a period of dry spell. They also developed a technique to decide on when the potential start of a wet season was true or false based on discriminating parameters such as, the number of wet days in the 20-day time slice preceding the potential start, and amount of rainfall in those 20 days. The same procedure is repeated in a scenario based on a 10-day time slice. Proud and Rasmussen (2011) suggested the calculation of the number of dry days near the start of the season to qualify for a false start. This requires a clear understanding of the start of season window. According to FAO (1980), the start of the growing season is the date when precipitation exceeds half of the potential evapotranspiration.

Investigation on the trends on the onset, cessation, dry spell, wet spell and number of rainy days is more beneficial than assessing changes in annual and seasonal totals across locations with high rainfall variability (Hadgu et al., 2013). This allows farmers to evaluate rainfall variability and directly relate it to their agricultural practices (Hadgu et al., 2013). Furthermore, correct timing of dry spell is crucial for ensuring successsful crop growth (Usman and Reason, 2004). Therefore, analysis of dry and wet spell is important in estimating the probability of intra-seasonal variability to support agricultural management practices.

In this study, we considered an onset of the rainy season to refer to the start of crops’ growing season. Eventually, Fsos is related to the false onset of the rainy season, a phenomenon linked to dry spell preceding the normal onset of the rainy season (Dunning et al., 2016; Sivakumar, 1988). The definition of false start has been based on a threshold approach to determine the actual onset of the rainy season, which is vital for rainfed farming. To predict the onset of rainy season, smallholder farmers in the SSA have over time developed local knowledge. Digging a hole to asses depth of soil moisture would also be informative on whether the crops can geminate when the seeds are planted. Since soils are anisotropic (or their properties such as, chemical, physical and biological properties, vary in all directions), digging holes to assess soil moisture may be deceptive. Farmers also tend to use phenological characteristics of existing permanent vegetation within their communities such as, trees, rangeland vegetation (for instance, “Opok” *Terminalia mollis* and Tamarind-*Tamarindus indica*-tree leaves regrowth) and changes in wind movement to signal the start of the season. Predicting the onset of rainy season based on local knowledge in most cases tends to be unreliable. Therefore, further efforts are being made to assess the variability in the onset of rainy season in order to identify the climate parameters that can be used effectively to explain the phenomenon. For instance, Reason et al. (2005) used Niño 3.4 to explain the false onset of the growing season in Limpopo South Africa, and the authors suggested that predictability of rainfall variability may be possible at a seasonal scale. Potential evapotranspiration and Inter Tropical Discontinuity (ITD) can also be used to explain the onset of seasons (Bello, 1997). Notably, most of these studies concentrated on the actual onset of the season and drivers of rainfall variability with limited information of the spatial temporal characteristics of Fsos. For instance, Camberlin and Okoola (2003), Jury (2018), Kansiime et al. (2013a) mainly paid attention to actual onset, seasonal variation and perception. Besides, the predictability of rainfall characteristics based on large-scale ocean-atmosphere interactions tend to be more plausible at regional than location-specific scale (Onyutha and Willems, 2017). Hence, further studies are required on the determination of drivers of rainfall variability at local scale with linkage to the onset of the growing season.

The aim of this study was specifically two-fold. Firstly, to map out areas within Uganda that have been prone to dry spell during the onset of the growing season. In this case the period 1999–2017 was selected based on data availability. The second contribution of this study was to detect and map out the timing, including the probability of the occurrence of the false start of the main growing season in Uganda. For the first objective of this study, Normalized Difference Vegetation Index (NDVI) time series data were used. The assumption here was that the NDVI could explicitly be used to identify variability at the start of the growing season, revealing its spatial temporal characteristics.

## 2 Materials and methods

### 2.1 Study area

**Figure 1:**
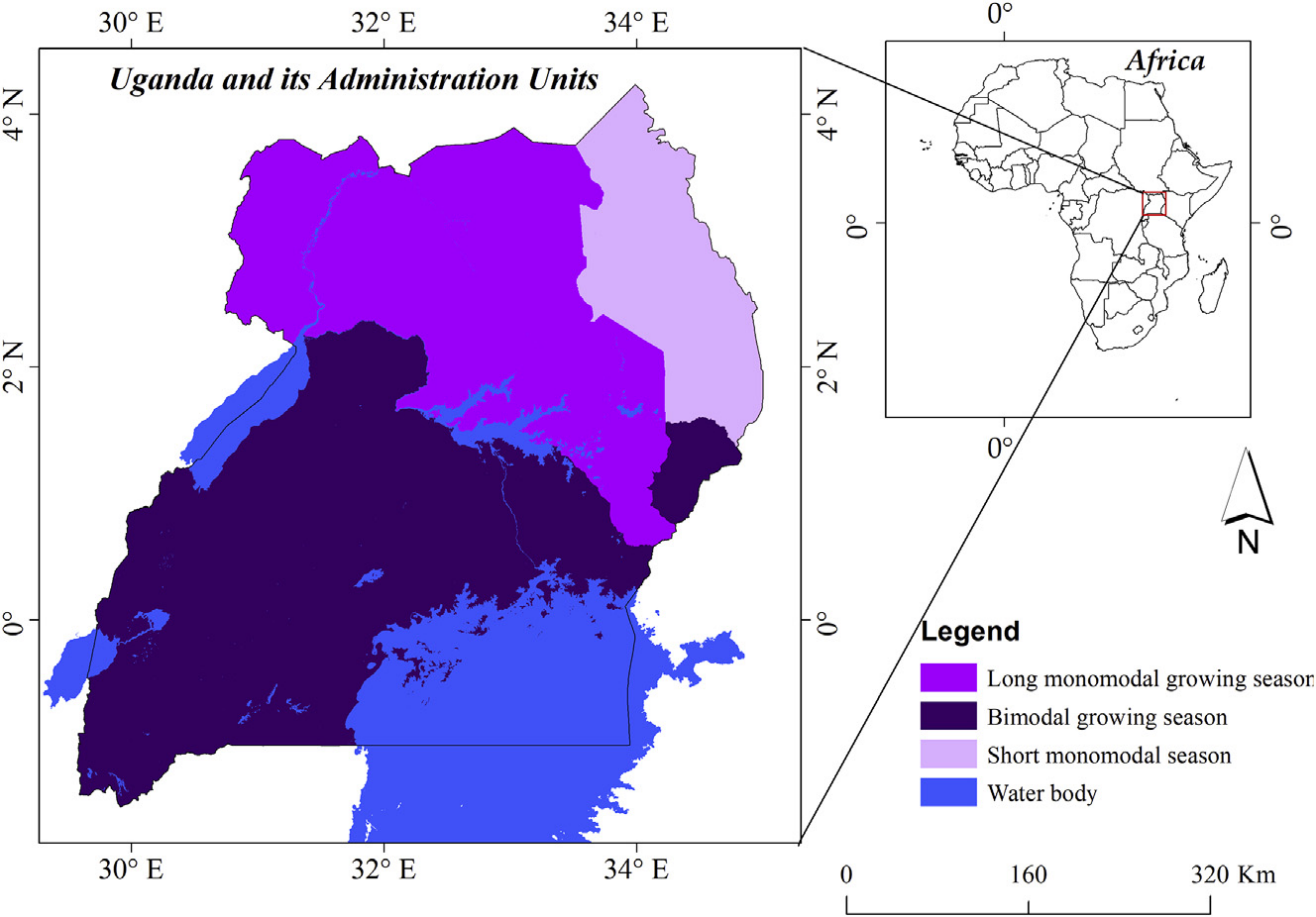
The characteristic of the growing season within the study area in relation to the climatic zones (Source: Majaliwa et al., 2015).

Uganda is a landlocked country located in the Eastern Africa. It lies between the latitude of 4^°^N and 2 ^º^S and longitudes 29^°^ W and 35^°^ E (Figure 1). It is bordered by Kenya, Tanzania, South Sudan, Democratic Republic of Congo, and Rwanda, to the East, South, North, West, and South West, respectively. Uganda has an estimated landmass of about 241,155Km^2^ and is rich in numerous natural resources such as forest, wetlands, freshwater (Lake Victoria, Lake Kyoga, Lake Albert, Lake Edward), mountains (Mt. Elgon, Mt. Rwenzori) and the Albertine rift valley along its western borders. The country slopes downwards from the southern towards the northern part of the Sudanese plain.

The climate of Uganda is naturally tropical, influenced by the Inter-Tropical Convergence Zone (ITCZ), subtropical anticyclones, moist westerly winds originating from Congo basin and the monsoon winds (Nsubuga et al., 2011). These forces, coupled with the contribution of the local geographical features such as large water bodies (Lakes), swamps, rivers, mountains interacting with the earth solar systems and interception of convective air, determine the existing weather patterns (Ogallo, 1989). Rainfall is triggered by the movement of air masses related to intercontinental convergence of the monsoon. Uganda being crossed by the Equator has the sun overhead it twice a year. The overhead passages of the sun, with a deviation of 4–6 weeks is linked to the onset variability of rainfall and distinction of seasonality type for different parts of the country (Asadullah et al., 2010). Eventually, Uganda has different climatic regimes shown by the variability in local temperature and rainfall (Majaliwa et al., 2015). Many regions of the country (especially those along the equator) bimodal rainfall pattern - March to May (MAM) and September to November (SON). Areas far north of the eEquator tend to receive rainfall characterized by a unimodal (long-term monomodal growing season and short term monomodal as characteristic of the north central-west and north eastern, respectively) pattern with the main rainy season occurring from June to August. The North eastern region of Uganda is semi-arid. Furthermore, there is variation across the country with respect to the timing, frequency, and distribution of the rainfall. Temperature also varies from one region to another. The amount of rainfall received is between 850 and 1700mm, while the temperature ranges from 16 - 30 °C. For this reason, Uganda is divided into sixteen climatic zones and nine Agro-Ecological Zones (AEZ) based on different agricultural farming systems dictated by different soil types, climate, landforms and socio-economic factors. Thus, as shown in Figure 1, different zones experience variation in seasonality and growing season (short-term monomodal, long monomodal and bimodal pattern) (Majaliwa et al., 2015).

### 2.2 Data and processing

**Figure 2:**
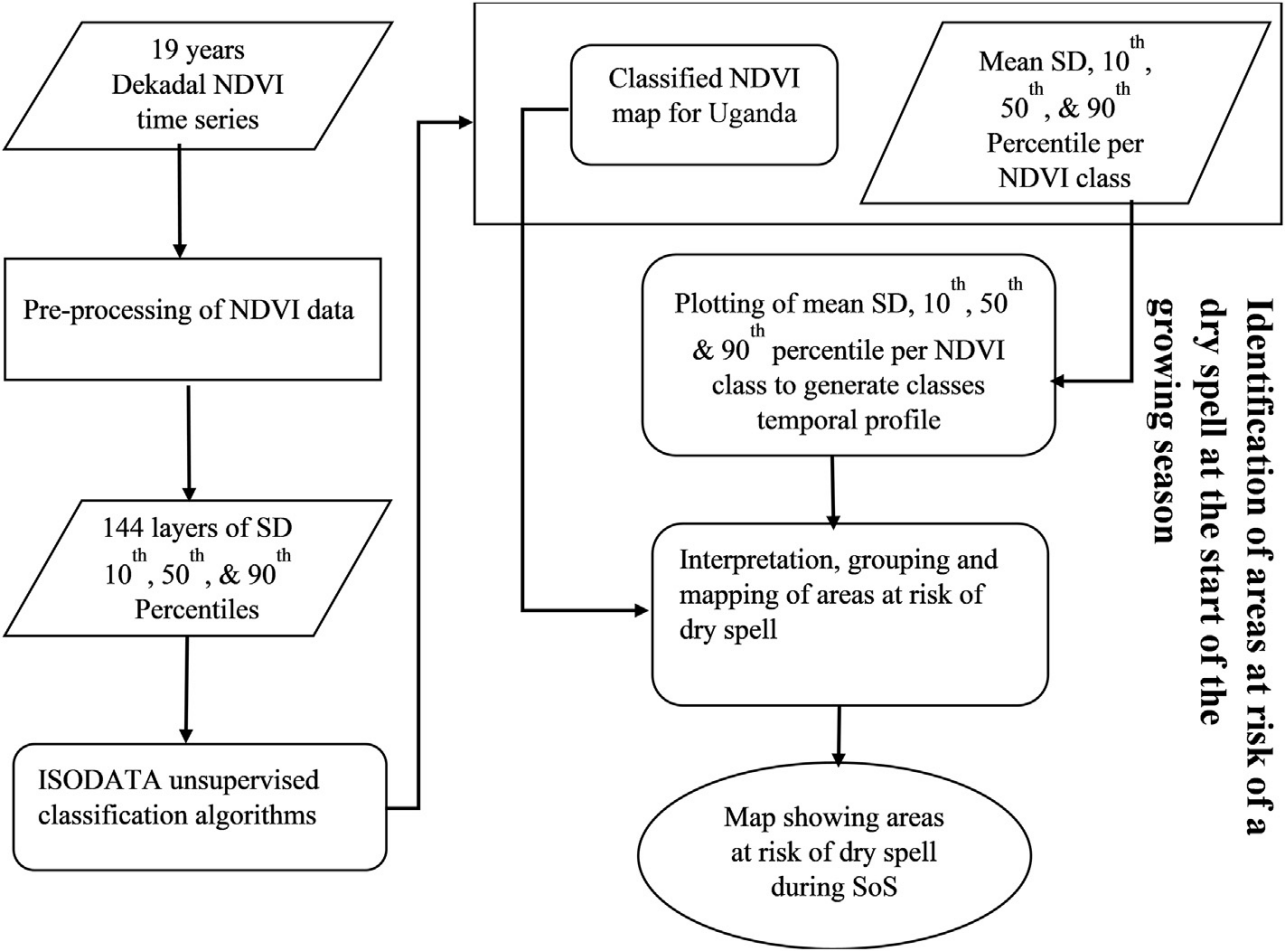
Flow diagram showing the preparation of NDVI dataset over 19-year period, in which the statistical parameters 10th, 50th, 90th & SD were extracted and used in the mapping of areas prone to dry spell during the start of the growing season.

Figure 2 provides key methodological steps in preparation of data used in mapping of areas prone to dry spell during the onset of the growing season. Furthermore, Table 1 summerizes information on the various datasets used in the this study.

**Table 1:** Summary of information on the different dataset used in the study.

Dataset	Description	Indicator	Source and Date of Access	Temporal coverage	Spatial Resolution	Data format
NDVI	Dekadal time series data of 1km by 1km resolution from November 1998 to February 2018	Vegetation performance	https://land.copernicus.eu/global/products/ndvi (Accessed on 25^th^ June 2018)	10days	1 km × 1 km	netCDF
CHIRPS	Dekadal and daily time series data of 5.5km by 5.5km resolution for the period 1999 to 2017	Rainfall estimates	ftp://ftp.chg.ucsb.edu/pub/org/chg/products/CHIRPS-2.0/ (Accessed: 22^nd^ October 2018)	Daily & Dekadal (10-day period)	Spatial resolution 0.05^0^ × 0.05	TIF
Land cover	Land cover data according to the global land cover classification system	Land cover types	http://www.fao.org/geonetwork/srv/en/main.home? (Accessed 05^th^ August 2018)	N/A	N/A	shp
Uganda Roads	Major and secondary Roads	Roads	https://download.geofabrik.de/africa/uganda.html. (Accessed:17^th^/September/2018).	N/A	N/A	shp
World Imagery	High resolution land cover data	Online access and saved as.img integrated in the map	ArcGIS baseline data (Accessed:17^th^/September/2018)	N/A	N/A	.img
Farmer Interview Data	Obtained from the field at pixel level with interviews conducted on farmer fields	Start of rainy season, cropping pattern	Farmers Interviewed	N/A	N/A	

#### 2.2.1 NDVI time series data for the period 1999 to 2017

SPOT (Système Pour l’Observation de la Terre) and PROBA-V missions NDVI 10-day time series (Table 1) were used for analyses. It is vital to note that the 10-day period is consistently denoted as dekad. The data adopted for this study was of the version 2.2 created from the top of canopy reflectance by the Flemish Technical and Research Institute (VITO) (Eklundh and Jönsson, 2015) and has been corrected for system errors and atmospheric conditions. Based on this correction, the product was deemed suitable for the purpose of this study. The values of NDVI here comprised the maximum value composite in a given dekad, and this was deemed beneficial since the effects of clouds were minimized, while other atmospheric effects also reduced, and thus, the product name “declouded image” (Chen et al., 2004; de Bie et al., 2011).

As a preliminary cleaning of the dataset, pixels of DN values 251–255, were removed, but the signal noise and spikes were still contributing to the unsmooth temporal profile (Table 2). This was followed by an application of the Savitzky-Golay Filter as explained by Eklundh and Jö nsson, 2017.

**Table 2:** The description of the values flagged off during preliminary NDVI data cleaning for the 19 year dekadal time series.

Flag value	Flag Name	Description
251	Missing	Error in RED/NIR
252	Cloud	Cloud/Shadow
253	Snow	Snow/ice
254	Sea	Water (Land Mask = 0)
255	Background	SM = 0

An unsupervised classification was performed on the 144 “SD-percentile-based” stacks of image using Iterative Self-Organizing Data Analysis Technique (ISODATA). Mean values of SD, 10^th^, 50^th,^ and 90^th^ percentiles were extracted per class to facilitate regrouping of the classes into areas prone to dry spell at the onset of the growing season.

#### 2.2.2 Climate hazards group InfraRed precipitation with stations (CHIRPS) dataset

In most areas across the SSA (where the case study area is located), observed weather stations are of low density (Onyutha, 2018b) poorly maintained, and most times out of use. Eventually, Satellite-based Rainfall Product (SRP) CHIRPS (Funk et al., 2014) was adopted in this study. This was because SRP provides spatialtemporal information even at ungauged locations and the data is of long-term spanning up to 35 years, thereby facilitating food security early warning studies, drought and flood monitoring and modelling. Besides, the use of SRP was to account for weather variability at a localized level.

CHIRPS products were developed with the ultimate aim of supporting assessment and monitoring of drought affecting the agricultural sector (agronomic drought), hence supporting the delivery of information relevant for food security early warning information systems. CHIRPS product was used to quantify hydrologic impact of decreasing precipitation and rising air temperature in the greater horn of Africa, concluding that, it has potential application in hydrologic forecasting and trend analysis in Southern Ethiopia (Funk et al., 2015). In East Africa (where Uganda is located), validation of CHIRPS rainfall estimates demonstrated a high correlation (*r* = 0.73) with gauge-based or observed data (Muthoni et al., 2018). Furthermore, CHIRPS products were applied in drought monitoring and hydrology related studies in the region (Agutu et al., 2017), Shukla et al. (2014). CHIRPS dekadal (10-day period) and monthly data compared with other satellite-derived products (TAMSAT3, the Integrated Multi-satellite Retrievals for GPM-IMERG, Climate Prediction Centre Morphing Technique-CMORPH, African Rainfall Climatology version two-ARC2 upon considerable analyses were found to perform better with pixel by pixel (0.73 ≤ *r* ≤ 0.87) than point to pixel (0.65 ≤ *r* ≤ 0.77) correlation (Dinku et al., 2018). The selection of CHIRPS product for this study was informed by these findings and its application for studies for areas within the East African region.

### 2.3 Method

#### 2.3.1 Identification of areas prone to dry spell at the start of the growing season

Several vegetation indicators and parameters derived from NDVI have been used to assess and study agronomic droughts. The most widely used indicator is the vegetative condition index. In this study, we relied on the NDVI anomaly information derived from the statistical parameters; 10^th^, 50^th^, 90^th^ and Standard Deviation (SD) (Figure 2) to detect areas that commonly experience dry spell during the onset of the growing season. It is vital to note that the use of SD does not assume normality. For a non-normality variance term we used the mean absolute difference (and outliers are less influential as it is based on distance apart not squared distances). By analyzing the temporal variation of the statistic parameter during window at the start of the season (5-12^th^ dekads for MAM and 20-27^th^ dekads for SON), we were able to catagorize different classes to give information on the risk of dry spell.

**Figure 3:**
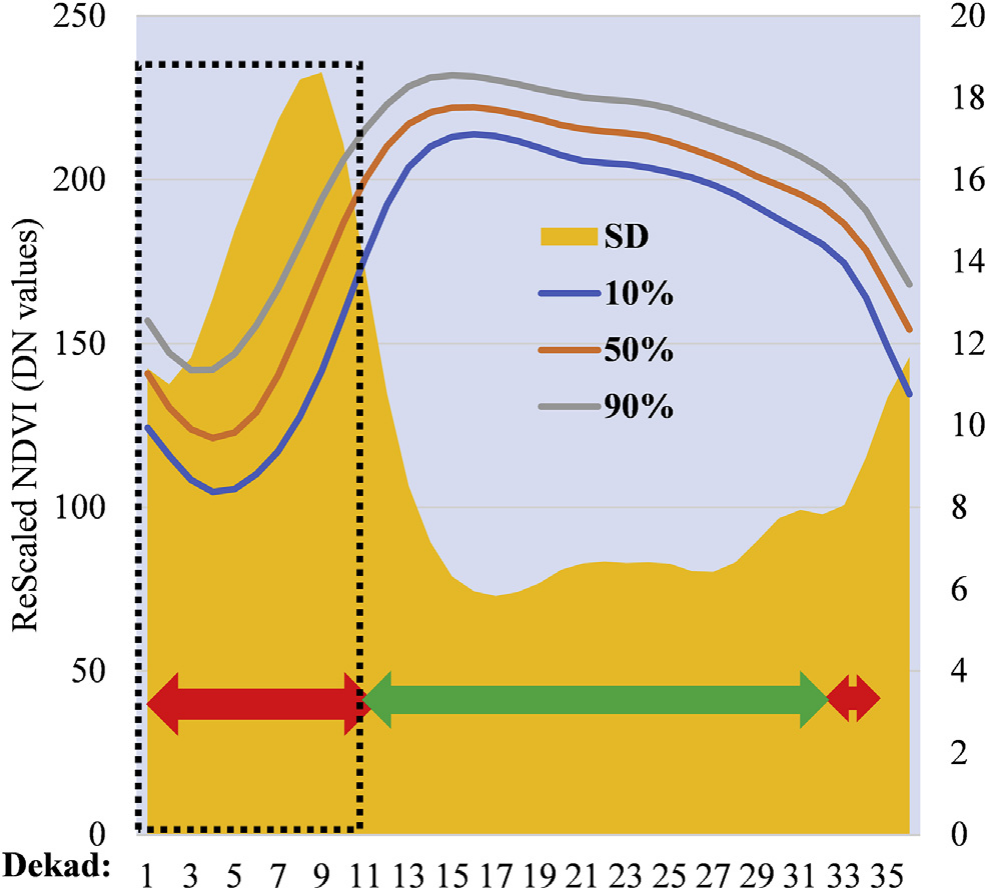
Framework for identification of areas at risk of dry spell at the onset of the growing season adopted from de Bie et al. (2008).

Based on the framework in Figure 3, by comparing the four parameters, where the peak of the SD concides with the start of season dekads, such a class is at risk while others are not. It was on this basis that the different clusters were grouped together and deemed to be at risk at the onset of MAM, SON or throughout the growing season. We relied on the successful application of a similar approach to develop an index-based insurance model in Ethiopia (Kees et al., 2018). The approach significantly relies on the information revealed by groups of pixels that exhibits similar characteristic to the variation in weather patterns. Because of the similar local climatic conditions such as short-term dry spell during the growing season, false start, the timing of drought, duration and gravity of its impact to vegetation, the groups of pixels have identical land cover and land use types (Kees et al., 2018). Meanwhile in another study (Yang et al., 2014), the 5^th^ and 95^th^ percentiles were used as climatological indicators in comparison with long times series in East Africa. Yang et al. (2014) used the gap existing between the SDs and percentiles (5^th^ and 95^th^) to demonstrate the occurrence of interannual variability in relation to MAM season. However, in this research, we included the SD as the basis of revealing risk information. Additionally, unlike the application by de Bie et al. (2008) to map out areas with different crop types and deriving cropping calendar, in this study we used a similar approach instead to map out areas at risk of a dry spell. The output from this process did not only allow mapping areas at risk but also aided visualization of seasonality differences and average changes of NDVI during the 36 dekads. Subsequently, the 10^th^, 50^th^, 90^th^ and SD statistics for each NDVI class for 19 years derived per dekad were extracted and plotted in excel revealing the temporal variations from the first dekad to the 36^th^ dekad. Comparison between the percentiles and the SD profiles was used to describe the onset of the season variability depicted by each class, thus, flagged to either be at risk of a dry spell or not. Subsequently, the temporal profiles revealed characteristics of the growing season within the different location and potential land cover types. This was crucial in aiding the decision to select which areas were characterized by annuals or perennials crops and those that had forest land cover. Finally, a map showing areas at risk of a dry spell during the onset of the growing season was created.

#### 2.3.2 Farmers perception on variability of the onset of the growing season

**Figure B1:**
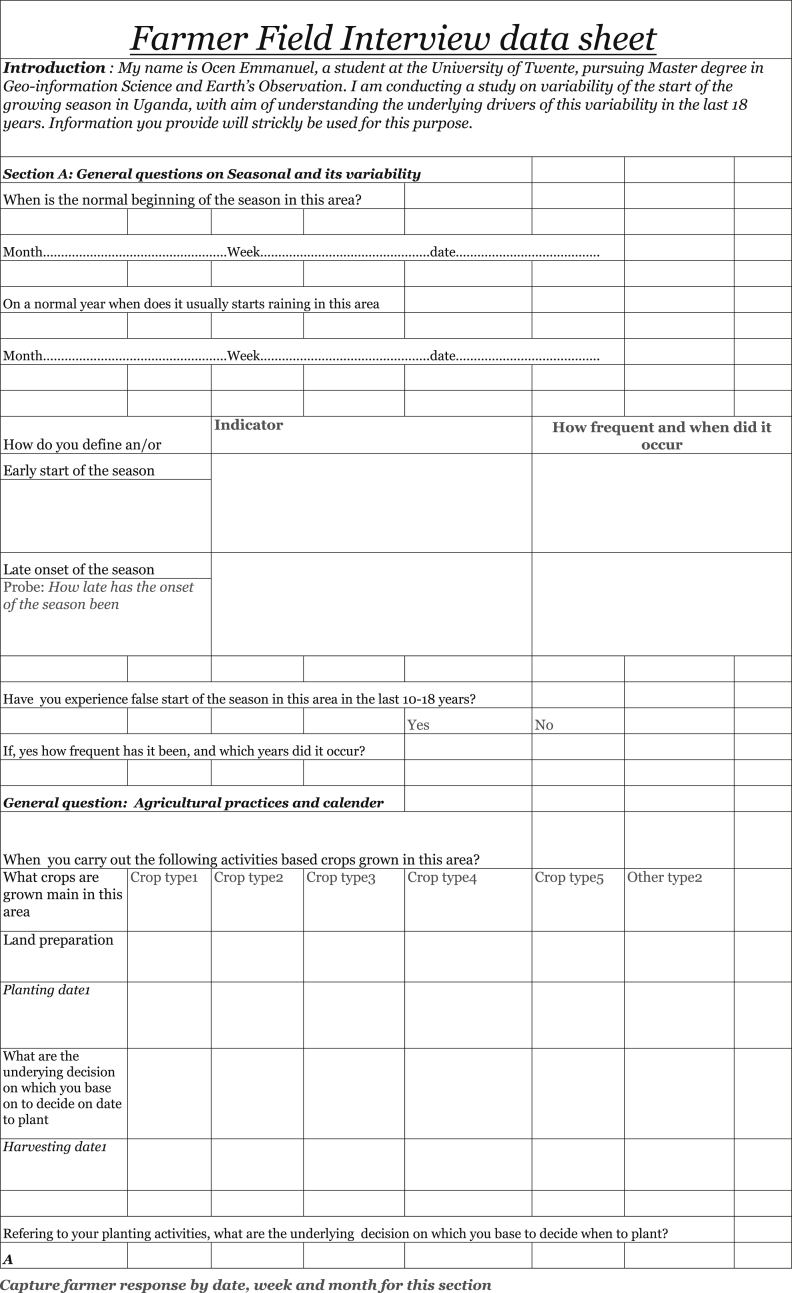
Section A of the interview question focusing on seasonality and its variability.

**Figure B2:**
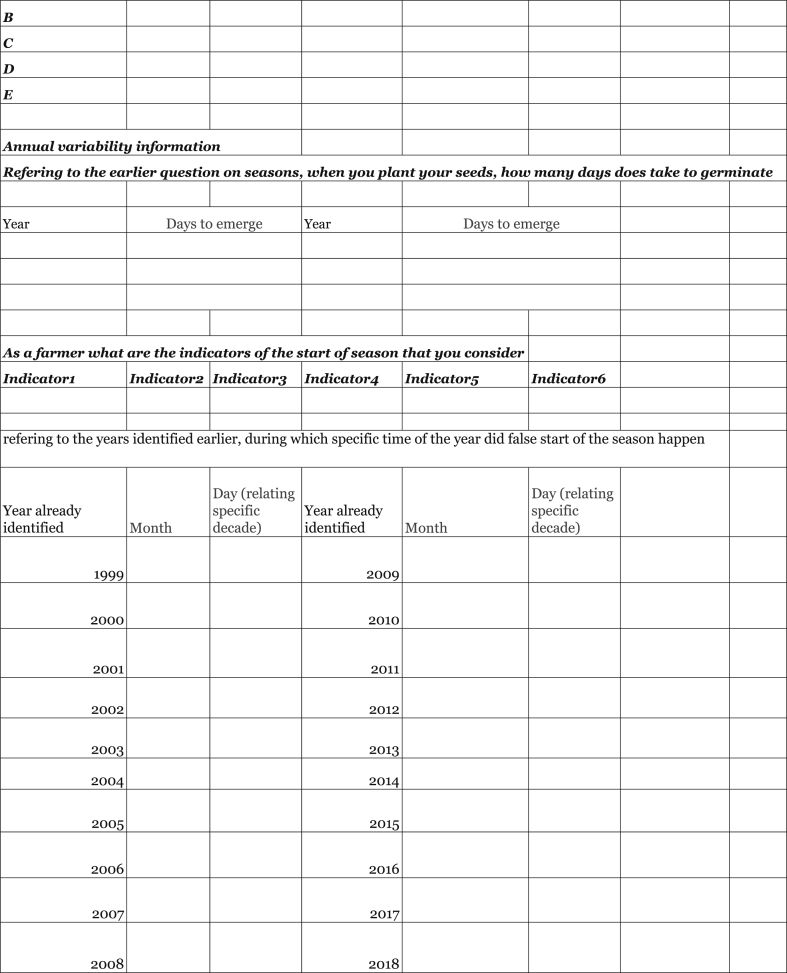
Section B of the interview questions focusing of existing agricultural practices and underlying factors informing such decisions.

In preparation to conduct field data collection, fourteen (14) pixels of arable cropping area were selected following the intersection of NDVI stratified map with land cover data. Linking stratified NDVI map to land cover types allowed the creation of classes with the different land cover types and their corresponding spatial coverage. This was vital to ensure the selection of relevant pixel in the context of this research (de Bie et al., 2011). The selection was based on the condition that the pixels were within at least 25km^2^ of arable cropland and furthermore characterized by risk of a dry spell during the start of the growing season. Field data characterizing farmers' perception and recall on onset variability were collected using designed interview schedule (Figure B1 and Figure B2).

Field data were collected through interviewing of farmers from 14 pixels each covering an area of 1 km^2^, located in the districts of Soroti, Kumi, Kole, Dokolo, and Pallisa. Initially designed questionnaire was pre-tested by being administered to 10 farmers in Pallisa. This allowed re-adjusting of questions to enhance validity of the required information. Finally, data were collected with the support of 6 field assistants and District Agricultural extension staff facilitating mobilization. A total of 72 adult farmers who had been practicing small-scale farming for at least 15 years, were available and willing to participate in the interview process. These criteria were adopted while considering the ability of the farmers to recall seasonality information, onset variability and long-term cropping practices that were relevant for the study, and duration for which field data was to be collected. This allowed retrieval of farmers’ historical insight on the onset of the growing season, which was considered critical to their decisions to commence cropping activities such as, ploughing and planting.

This information based on farmers' experience about the growing seasonal variability, such as the definition/distinction between the start of the growing season and false start of the growing season was vital to understand farmers' perception on seasonal variability. Farmers' knowledge on the onset of the season variability was clustered into awareness of early, normal, late start and the false start of the season. The obtained information was analyzed in general terms for the 19 years, evaluating farmers' definition of SoS, long term farmers’ recalls of weather-related variability and specifically for the year with vivid recall (2015–2017). The data were analyzed at a pixel level, thus, facilitating integration with remotely sensed data. Information derived from the field survey were explored and analyzed. The analysis of farmers' responses, was to help identify relevant parameters that could support the definition of the Fsos and subsequently allowing for determination, quantification and mapping of this onset variability.

#### 2.3.3 Detection and mapping of the spatial temporal variability of the Fsos

We integrated information obtained from farmers' through field interview and the data from remote sensing products. Three-year climatological time series obtained from CHIRPS and NDVI values for the surveyed pixels were analyzed and compared with information that farmers could recall and their perception on the growing seasonal variabilities. The choice of these years was based on the ability of farmers to recall anomaly information and their cropping activities during these years. in order to capture the information that coud easily be recalled by farmers, we started from 2018 and went backwards in time till when the respondent could not remember adequately anymore. We found out that all 72 respondents could adequately recall and provide complete information over the latest 3-year period (2015–2017). Information provided by farmers relating to the onset of the rainy season, planting date and a false start was annotated on the graph for all the surveyed pixels. Such a qualitative approach allowed comparison of what the farmers reported and the information revealed by both NDVI and CHIRPS products. Possible disagreement, such as, wrong detection of Fsos in the years 2015 and 2017, prompted further investigation on what could possibly have influenced the farmers' recall or what other factors affected crop production that prompted the farmers to identify the period to be characterized by an Fsos.While comparing the data, attention was placed on the identification of SoS in relation Fsos reported date by the farmers. This process facilitated detection of first rainfall peak and related numbers of rainy days corresponding to this peak thereby leading adjustment of the definition of SoS in view of detecting the occurrence of a false start. Total accumulated rainfall and rainy days (RD) per dekad other than rainfall events were used to arrive at the definition of Fsos. Accordingly, the definition by Sivakumar (1988) was modified to factor in rainy days apart from considering only the rainfall total. The modification was informed by field survey data, where the number of rainy days to the farmers was more important, thereby allowing for the distinction between the true and false start of the growing season. Subsequently, this study considered an Fsos to be a dekad (dk_n)_ after the 5^th^ with atleast two rainy days and the subsequent dekad registering zero rainy days. The rainy days in this study was taken to be a day with total accumulated rainfall >5mm. This considered evapotranspiration rate, annual crops’ water requirement at initial stage of growth and rain water depletion fraction in the soil (Kyagulanyi et al., 2016). Where an average potential evapotranspiration (ETo) for Uganda is 5mm, the mean depletion fraction is around 0.4 during initial growth stages of crops. Furthermore, the occurrence of at least two rainy days would mislead the farmers to think a rainy season has started thereby prompting the planting of crops.

Condition defining the Fsos was applied to all the pixels under consideration. This, however, excluded pixel of water bodies (Lakes, Rivers and stream) that were masked out prior to an application of the relevant Fsos-related condition. A total of 6818 pixels were considered over the 19-year data. The SoS was first identified and checked against the 2^nd^ condition and flagged either as true SoS or Fsos. The corresponding rainy days associated with the identified Fsos were selected. Furthermore, we checked the mean accumulated rainfall for each Fsos date and ensured all the pixels had rainfall amount not less than10mm.

With these aspects identified for each pixel using the 19-year data, we determined (i) the probability of occurrence of Fsos for each pixel as a measure of risk, (ii) the mean number of RD relating to the Fsos and compared with agricultural areas. A combination of these two defined spatially where the risk and impact associated with Fsos were higher than those based on the information provided by the farmers.

## 3 Results and discussion

### 3.1 Mapping of areas within Uganda at risk of a dry spell at the start of the growing season

#### 3.1.1 ISODATA NDVI classification

**Figure 4:**
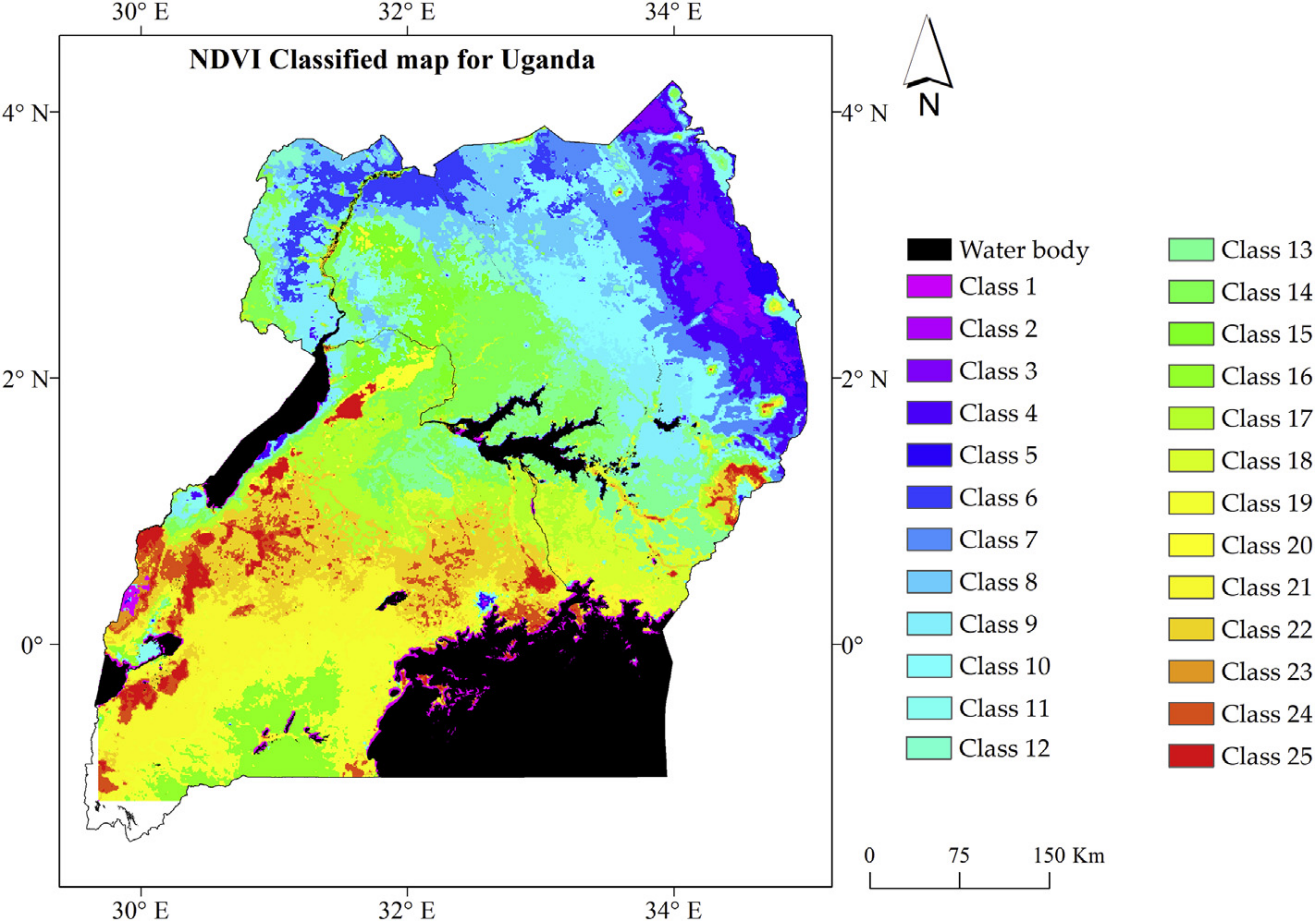
Spatial variability of Uganda land cover for the year 1999–2017 as depicted by the unsupervised classification results. The classes contain mixed land cover types with different spatial temporal characteristics.

Results from the ISODATA classification yielded 25 classes in the NDVI stratified map (Figure 4) on the 144 data layers that comprised the 10^th^, 90^th^, median and SD values. The analysis, derived 19-year spatial temporal dynamics of the land cover for Uganda obtained by extracting the temporal profile per class, generated from cluster signature data saved in ERDAS. Through visualization, we were able to infer the seasonality characteristics across the landmass, indicating bimodal versus unimodal short and long term single growing season. This provided insights on variation and characteristics of vegetation cover types over the last 19 years which were verified from the field during the farmers' interviews in the areas within the selected pixels.

In Figure 4 and Figure A1, B1 and B2, C1 we noted that the classes were ordered according to their mean NDVI. Classes 1–5 corresponded to bare soil or areas with sparse vegetation cover, most of which were in the semi-arid part of the study area and were characterize by unimodal growing season. Classes 6–10, 12–14, 15, 17, 18 and 20 are areas with green vegetation cover with long term unimodal growing season in a year as also found by Jameson and MacCallum (1970) in which they stated that the northern part of Uganda is characterized by short period of dry spell in June–July 1^st^ to 2^nd^ season transition. Thus, the NDVI values do not fall to a minimum value since planting is done immediately following the 2^nd^ season rainfall onset, hence, the unimodal long growing season. On the other hand, classes 11, 16, 19, 21 and 22, corresponded to areas with bimodal rainy season with two clearly separated green up and decay period in a given year. We observed that for classes 23–25, the temporal profile showed constantly high values of NDVI throughout the year. This corresponded to areas with dense vegetation that are ever green.

Additionally, we noted that the NDVI annual temporal profile differed distinctively from one year to another in both minimum value and amplitude. This indicated the annual variability within the different classes that were displayed by the profile. The onset window from 7^th^ to 14^th^ dekad revealed the same information indicating an early start, (a late start in some years) and hints on the possibility of a false start in a particular year. Nevertheless, this approach may not yield conclusive information on the occurrence of Fsos for specific areas without incorporating meteorological data.

The approach demonstrated that image classification can be performed using derived statistical parameters of SD and percentile, generating important information such as, variability in the onset of the season, seasonality variation, and annual variability as also revealed by the timing of the first increase of NDVI from the minimum in a given year. A similar approach was applied by Höpfner and Scherer (2011) in Morocco to derive information on intra-annual and inter-annual variation in vegetation. This approach was considered a stable classifier, thus, justifying the effectiveness of applying the relevant parameters. Meanwhile, Gasmi et al. (2016) while using ASTER dataset for geological mapping applied principal component analysis to reduce 9 bands of ASTER for comparison with the reference geological map. Application of the principal component analysis was to reduce the volume of data for analysis. The main disadvantage of the principal component analysis in image analysis lies in the difficulty to accurately evaluate the covariance matrix involved (Phillips et al., 2005). In this study, the use of statistical parameter was to address the problems of large data dimensionality in the long-term time series to shorten the computational time while revealing the relevant required information.

From this study, we can infer that NDVI has a strength and capability in describing the variability during the growing season in relation to weather and climatic changes that affect vegetation performance and consequently reveal information captured through remote sensing. The advantage of this approach is the ability to generate the temporal information from which further characterization can be made (de Bie et al., 2011).

#### 3.1.2 Areas at risk of a dry spell at the beginning of the growing season

**Figure 5:**
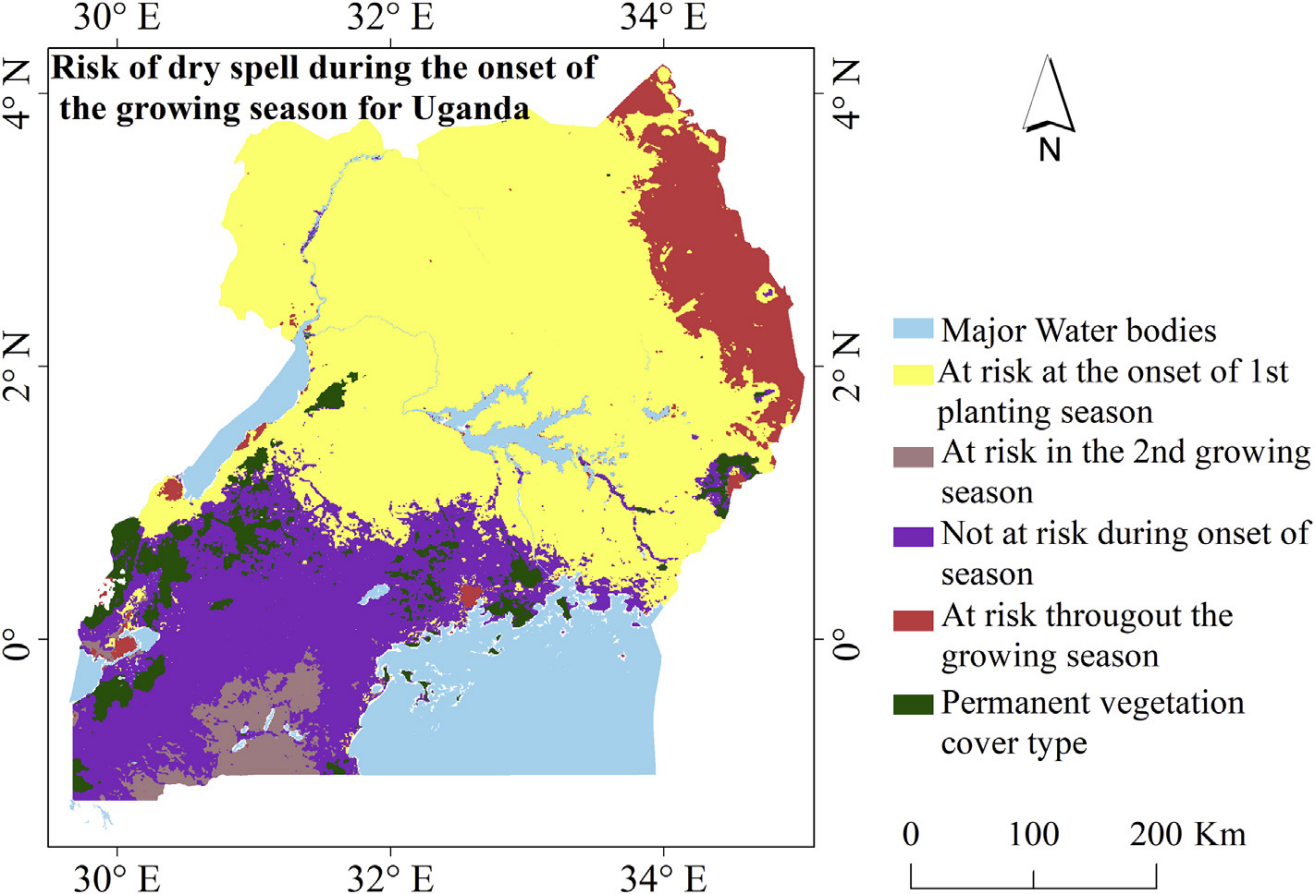
Spatial coverage of distinct categories of areas at risk of a dry spell during start of season.

Our analysis revealed that most of the regions within Uganda were at risk of a dry spell during the beginning of the growing season for the study window, especially at the onset of the main growing season (MAM), except in the south western region that revealed risk during the 2^nd^ planting season (SON) as seen in Figure 5.

Based on seasonality, a total of 12 classes of pseudo bimodal rainfall regime were found to be at risk at the onset of MAM season and this corresponded to 58.1% of cropland areas. Meanwhile, 5 classes were at risk at the onset of both MAM and SON corresponding to 8.8% of the cropland area. These areas cover the semi-arid region of Uganda often experiencing occasional dry spell not only at the onset of the season but all through the year. Nakalembe (2018) observed that farmers follow their cropping calendar and once seeds are sown, many are not able to replant if rains fail to fall at the start of the season. Therefore, the occurrence of a dry spell in this region can severely impact on agricultural productivity.

Only one profile with 3.7% of cropland area in the south western region indicated risk at the onset of SON. This is consistent with the results of the Cooper and Wheeler (2017) in which farmers revealed that in recent years they had experienced variability in the SoS in SON season, pointing out increasing uncertainty in relation to false start. Surprisingly, three classes with 23.2% of the cropland area did not show a risk of a dry spell at the onset of the growing season. However, this is likely not the case; perhaps it is because of the existing cropping system in this region of Banana-Coffee, the mean NDVI will tend to remain relatively less variable since the perennial crops would have an effect. Conducting field surveys and use of gauge station data would allow for validation of this finding, hence proving the application of this model in drought monitoring assessment. The other classes mainly of permanent vegetation cover (6.2%) did not indicate the risk of dry spell throughout the year.

This finding obviously does not imply that the dry spell is experienced at the same period in all areas but provides evidence that within the last 19 years, they were at risk during the onset of the season. This dry spell could be attributed to Fsos or an extending dry spell into the growing season resulting in the late start of the growing season. The latter has been reported by Jury (2018) pointing out 2005 and 2009 as the affected years in the Lake Kyoga basin. Similar findings have been documented in recent studies (Cooper and Wheeler, 2017; Kansiime et al., 2013b; Mugume et al., 2016; Nakalembe, 2018; Orlove et al., 2010) pointing to the facts of a dry spell during the growing season, with the latter mainly characterizing prolonged dry experienced in Karamoja region.

**Figure 6:**
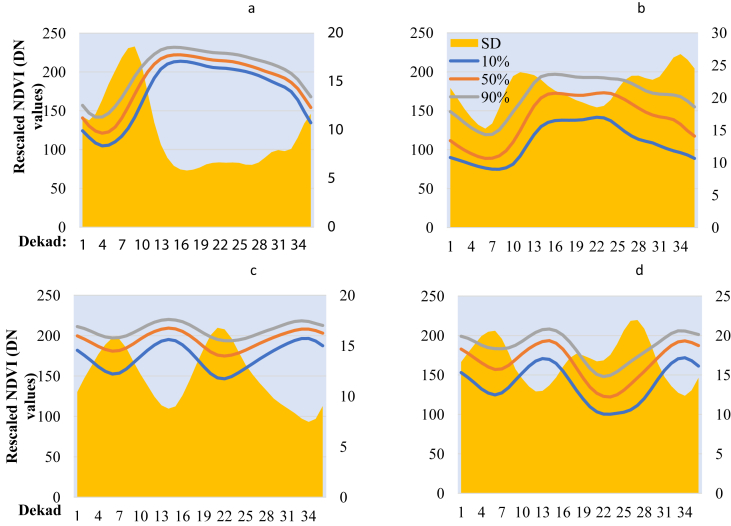
NDVI profile for (a) areas at the risk of dry spell of MAM growing season, (b) the entire year, (c) not at risk, and (d) areas at the risk in SON growing season.

Specifically, for areas at risk of dry spell, the mean SD as seen in Figure 6 was high at the onset of growing season indicating variability within this temporal window. For areas not at risk, the SD was high during the dry season, i.e., in dekad 7-11 (March–April) for unimodal and 26–27 (September) which are a window for start of the growing season in Uganda, and the 34-36^th^ dekad in the dry season. The application of SD in this study is similar to the one used by Hall-Beyer (2012) to quantify the inter-annual variability over the 25 year period in Canada and just like in this study the mean value for SD for specific areas were used, thus, appropriate for mapping areas at risk.

Overall, dry spell occurring during the start of the growing season has a substantial negative impact on the general productivity of the agricultural sector due to reduced yields and low production among the farming community. The implication is that, correct detection of SoS by farmers and stakeholders delivering forecast information would translate into a successful season and vice versa.

### 3.2 Characterization of the false start of the main growing season (1999–2017)

#### 3.2.1 Farmers experience and perception on the start of the growing season

**Figure 7:**
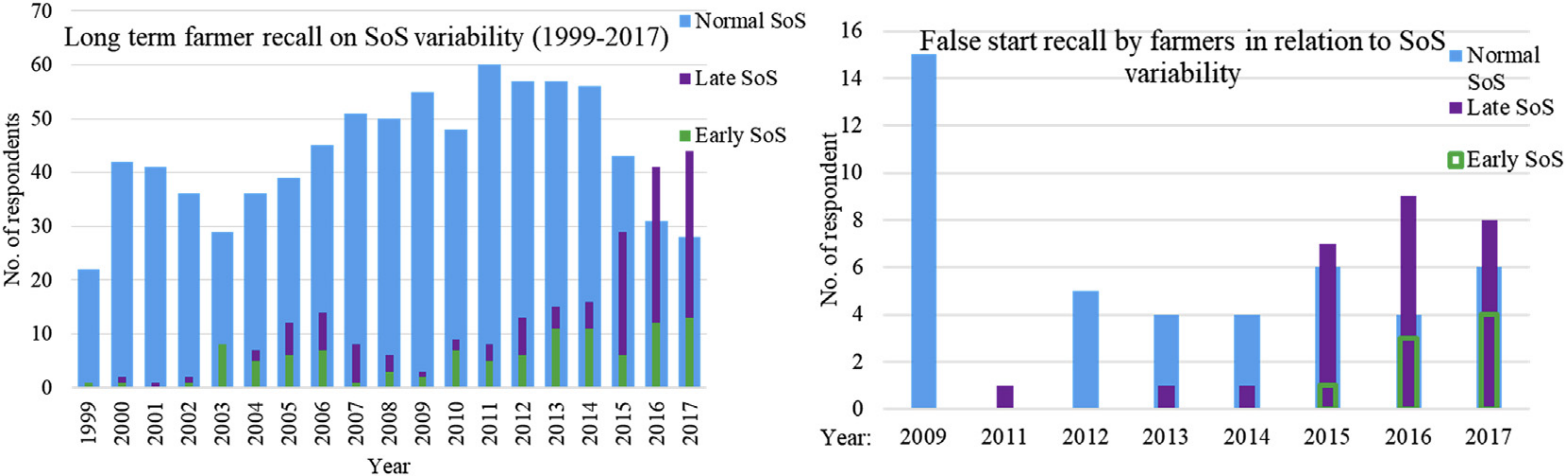
a) Long term farmer recall of onset variability and b) the false start of the season.

The farmers' perception revealed that the start of the season generally for the last 19 years had been variable from late, normal, early and sometimes associated with a false start (Figure 7). Atleast 15% of the farmers reported that they experienced an early onset of the growing season, from 2013 - 2017. About 40% of the farmers reported that over the period 2015–2017, there were late onsets of the growing seasons. Both farmers could have been correct in their recall of onset of the growing season. For instance, if the rains started early and dry spell sets in, some farmers would identify it as early SoS. On the other hand, farmers would have correctly detected it as an Fsos if the dry spell period was long. Thus, such a year would definitely be reported by the farmers to have been characterized by a late SoS. Thus, an important note to be taken is that late onset does not necessarily mean there was a false start of the season. Furthermore, results indicate that the median for the SoS as identified by farmers for both long-term and short-period recalls was dekad 8. This was consistent with the results from an analysis conducted using data from the closest rainfall gauge station at Serere. In a study Botai and Combrinck (2012) indicated 16^th^ pentad as median onset and planting window 12^th^–20^th^ pentad (6–10^th^ dekad). These findings are inferred in the NDVI SoS-based information where the mean onset date was the 9^th^ dekad for the 14 pixels surveyed indicating vegetation development after the onset of the rainy season. Additionally, during this window, the sun is overhead the Equator triggering radiation impact on land surface and atmospheric clouds thereby leading to formation of rainfall.

In a related study conducted by Kansiime et al., 2013a, Kansiime et al., 2013b in Lake Kyoga basin, the farmers reported that they had late onset of the season in recent years emphasizing that the first planting season (or MAM rainfall) had its onset shifted from the early March to late March. Consistently, this study showed that from the 2005 to 2017, farmers in Soroti and Kumi reported the late onset of the MAM season. For 2012, 2014, 2015, and 2017, 25% of farmers in these areas reported late onset, 17% cited an early start while 58% indicated that the seasons were typical. The farmers further pointed out that there was an increasing problem of a dry spell during the start of the seasons and sometimes flash floods in other years thereby suggesting an increased risk during the production period over the years.

We further noted that from the 14 pixels surveyed in the districts of Soroti, Kumi, Pallisa, Dokolo, Oyam and Kole, there were variations in the way farmers experienced the onset of the season. Analysis based on pixels of CHIRPS for SoS also indicated an early onset as 6^th^ and late SoS as the 11^th^ dekad and SD of 1 dekad for the MAM season. Accordingly, the perception of farmers on variability on the SoS as identified in this study were in tandem with related work on the same subject. For instance, in studies conducted in Uganda, farmers, perception indicated that increasing temperature and seasonal variation made the first growing season unreliable and less favourable for crop production (Osbahr et al., 2011; Okonya et al., 2013). Other studies in South Africa assessed the perception of farmers in relation to onset, duration and cessation exploring the degree and frequency of characterized variability (Reason et al., 2005; Simelton et al., 2013). And, the farmers reported shift in the onset and that it was becoming unpredictable, highlighting the occurrence dry spell after planting. This is similar to studies conducted in West Africa by Laux et al. (2009); Odenkunle (2004); Sobowale et al. (2016).

Rainfall variability has a direct impact on crop productivity. The late onset of the season results into shortening of the growing season; therefore, only annual crops with short growing periods can be grown. In this study, we noted that dry spell after planting was damaging to the crops because almost immediately after sowing the rains would stop and the seeds could not germinate and grow as expected. Occurrence of dry spell at the beginning of the growing season can lead to an overall low crop productivity (Salack et al., 2015). Evidently, from this study, especially in 2016 only two out of 13 farmers reported that they were affected by Fsos, and these farmers actually undertook replanting. This made analysis in relation to replanting impossible as several farmers that were reportedly affected did not replant.

In relation to the seasonality information, it was inferred that there could be a relationship between the cropping practices among farmers from different areas. This is not surprising as the agro-ecological zoning includes an aspect of local climatic variability in its delineation, thus the existing cropping practice. Two facts were important to take note of. Firstly, across the different districts, land preparation tends to commence prior to the onset of the season and planting follows after the onset. Secondly, different areas have different major crops, therefore suffer from the impacts relating of Fsos to varying extents. The differences in the crops planted tend to be determined by the local climatic condition, elevation, slope and soil characteristic, where certain types of crops will thrive better than others*.*

#### 3.2.2 Spatial temporal characteristics of a false start in Uganda

**Figure 8:**
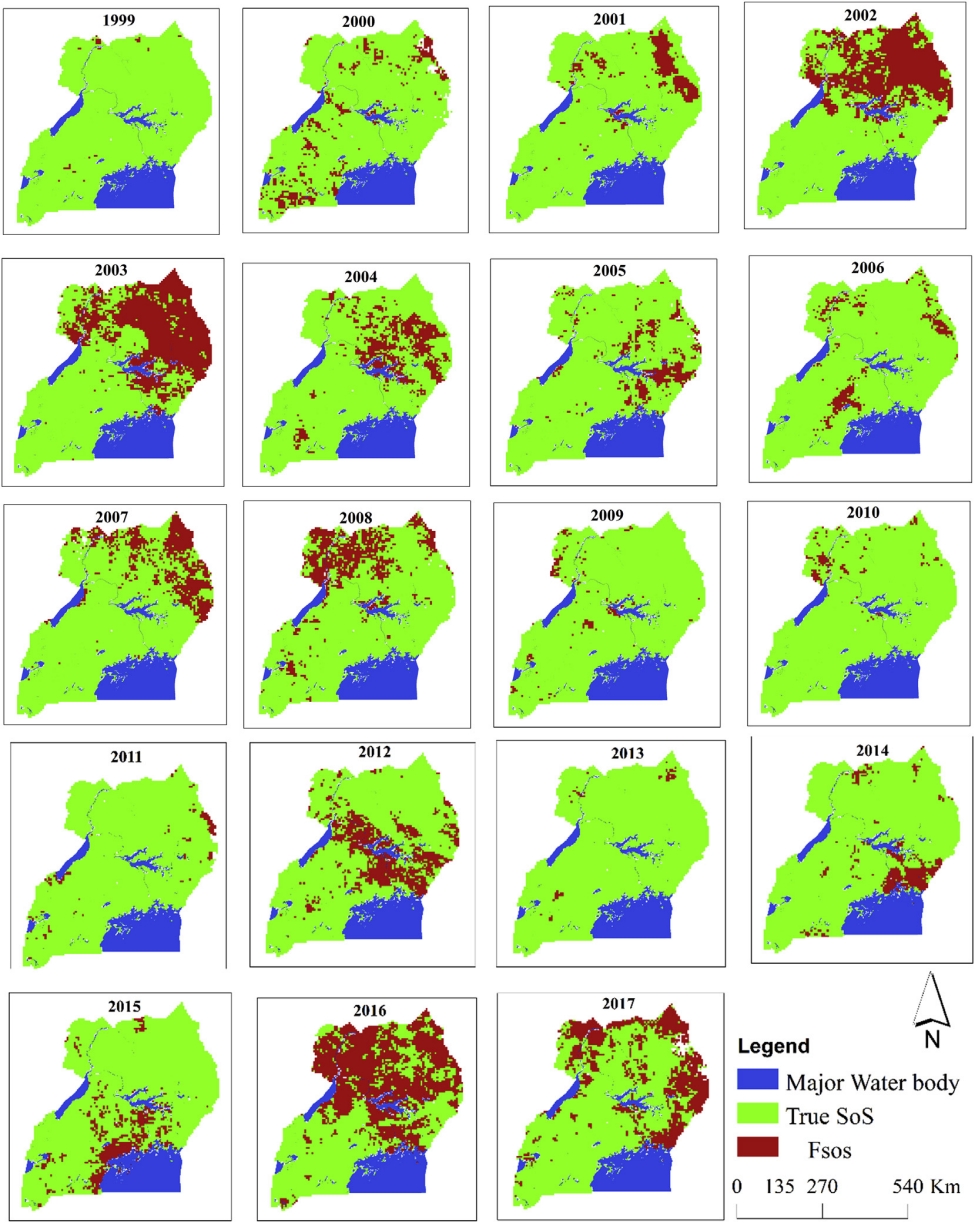
Spatial extent of the occurrence of the false start of the main growing season from 1999 to 2017.

Table 3 and Figure 8 generally reveals that, all the years were affected by the false start of the main growing season, with the spatial extent varying from one year to another. Proportion of pixels affected by Fsos in 2002, 2003 and 2016 were 32%, 37% and 46%, respectively. Meanwhile, few pixels were affected by Fsos in 1999, 2009, 2010, 2011 and 2013. Focusing on the dates in Table 4 the findings indicate that false start is independent of the timing of SoS, it can occur following early, normal or late SoS. Our analysis revealed that majority of pixels affected by Fsos had SoS 1^st^ date between the 6 to 9^th^ dekad, with the year 2002 event relating to 7^th^ dekad, while 2003 in 9^th^ and 2016 in 7^th^ for most pixels. Hence, it is highly likely that farmers can easily be duped into planting. Consequently, the years characterized by Fsos can possibly result in late SoS in a given area. The late start of the season may also be due to an extended dry spell into the start of the growing season in a given year.

**Table 3:** The percentage of the total number of pixels affect by Fsos covering the arable land cover for Uganda.

**Years**	No. of pixels			Proportion in Percentage	
	**FSoS**	**SoS**	**Total**	**Fsos**	**SoS**
1999	38	6780	6818	1%	99%
2000	603	6122	6725	9%	91%
2001	525	6291	6816	8%	92%
2002	2203	4615	6818	32%	68%
2003	2550	4268	6818	37%	63%
2004	1007	5811	6818	15%	85%
2005	611	6182	6793	9%	91%
2006	404	6414	6818	6%	94%
2007	1062	5730	6792	16%	84%
2008	1164	5645	6809	17%	83%
2009	178	6637	6815	3%	97%
2010	168	6650	6818	2%	98%
2011	141	6652	6793	2%	98%
2012	1292	5526	6818	19%	81%
2013	65	6753	6818	1%	99%
2014	491	6324	6815	7%	93%
2015	706	6112	6818	10%	90%
2016	3153	3665	6818	46%	54%
2017	1736	5001	6737	26%	74%

**Table 4:** Dekads in the 19 years frequently associated with a false start.

**Year**	**Dekad/Number of pixels affected**						
	**6**	**7**	**8**	**9**	**10**	**11**	**Total**
2002	0	2187	1	15	0	0	2203
2003	864	19	0	1667	0	0	2550
2007	49	8	997	7	1	0	1062
2008	0	113	0	1051	0	0	1164
2012	4	1234	54	0	0	0	1292
2016	0	3153	0	0	0	0	3153
2017	1353	16	0	367	0	0	1736

**Figure 9:**
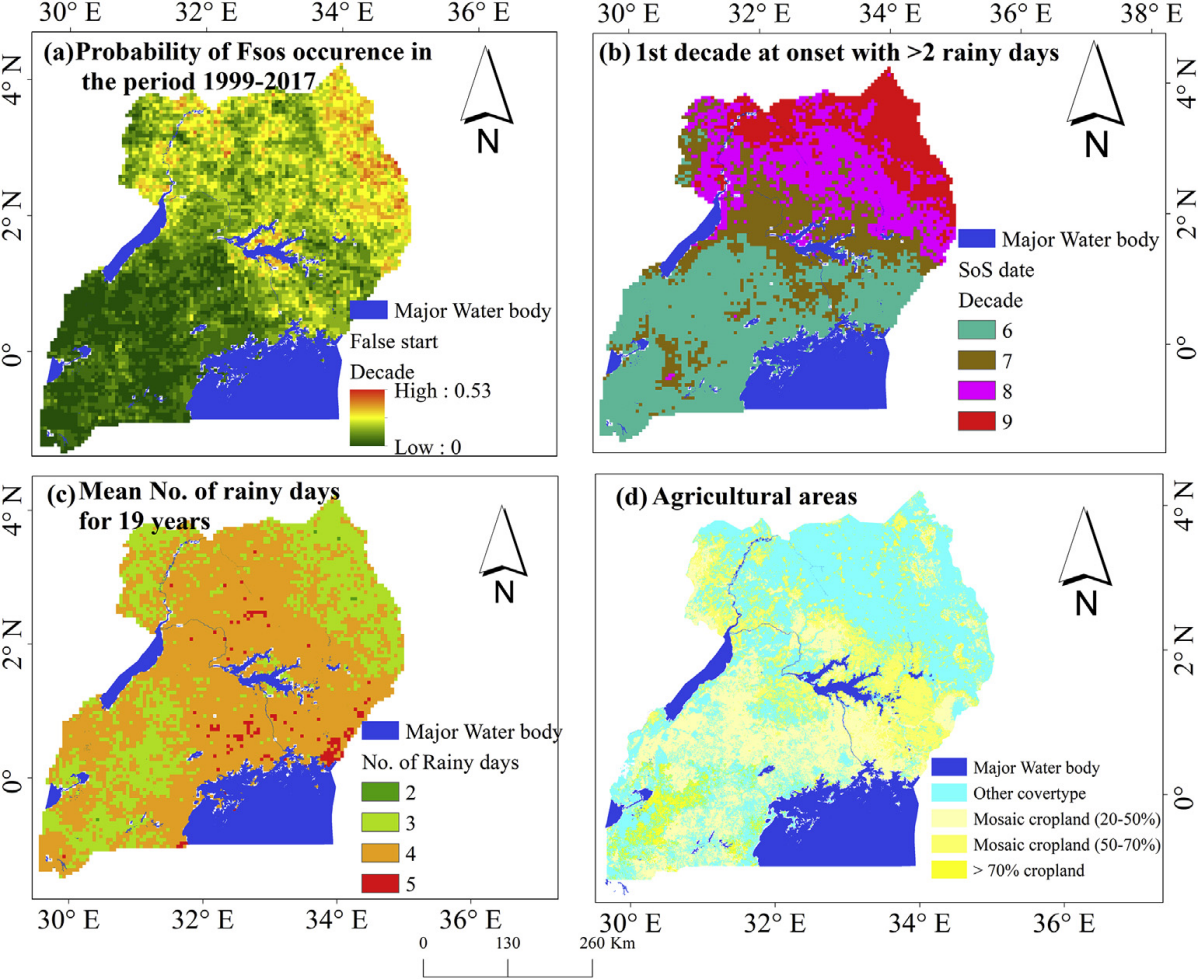
(a) the probability of false start occurrence, the probability of false start of the season increases from green to red. (b) its associated rainy days, (c) dates in dekad compared to onset of the season (d) the arable cropping areas in Uganda as an indication of vulnerability and impact of risk to farmers.

Furthermore, as shown in Figure 9, we observed that the probability of occurrence of Fsos was mainly high in the Northern region. However, the highest probability of occurrences was in the North Eastern part of the country constituting the semi-arid Karamoja region (characterized by Fsos occurring at least 10 times in the 19 years, *p* = 0.53). This suggested that the farmers within this region are often confronted with the risk from dry spell. Also, it is important to note that in this region (with higher probability of Fsos) the major crops planted in the MAM season include maize, millet, cassava, soybeans, sunflower and groundnuts. These crops vary in their sensitivity to heat and moisture stress, thus, the likelihood of failure to germinate or dry up after germination (in case of Fsos) will also vary from one crop to another. The leguminous crops such as beans, soybeans and oilseed sunflower crops are more vulnerable to the occurrence of Fsos right after planting because of their less tolerance to heat stress compared to the cereal crops such as maize (Sita et al., 2017; Nadeem et al., 2019). Hence, farmers should cautiously monitor the start of the rain and avoid rushing into planting of leguminous crops.

Additionally, when the start of the rainy season in the 7-9^th^ dekad following 4-rainy days is associated with Fsos, the risk becomes higher as 4RD pushes farmers into planting of crops. Furthermore, the areas around the major water bodies indicate a relatively high probability of Fsos. This is probably due to the relation between seasonal variation and the influences of lakes on local climate. The seasonal rainfall amount tends to decrease with distance from the lakes. In this line, areas around the Lake Victoria and Kyoga are often characterized by higher rainfall amounts than those over other parts of the country.

Coincidentally, the areas with frequent Fsos shown in Figure 5 were at risk of a dry spell during the SoS. This suggested that the risk identified in section 3.1.2 may possibly be associated with the Fsos. The Fsos is possibly due to the early arrival of Inter-Tropical Convergence Zone (ITCZ) that results in an early onset followed by a quick cessation whose drivers to this point seem unclear. While migrating northwards and southwards, the ITCZ's northern and southern boundary might be linked to the occurrences of the Fsos during the first and second growing seasons, respectively. This, though not examined in this study, may be experimentally investigated using regional climate models which suitably reproduce spatial temporal variation in the observed rainfall statistics across the study area. Furthermore, the ITCZ is closely related to the meridional sea surface temperature (Freitas et al., 2017). The linkage of the Indian Ocean Dipole (IOD) to the El nino southern oscillation (ENSO) is potentially influenced by the ITCZ southern boundary (Freitas et al., 2017). The fact that only about 30% of the IOD events occur independently of the ENSO events indicates the need to take into account the IOD-ENSO relationship (Onyutha, 2020) in investigating how the spatial temporal variation in the Fsos can be driven by large-scale ocean-atmosphere conditions. The ITCZ has a significant influence on the onset and duration of rainfall in Uganda, with the movement towards the north during the MAM season. Because of its sensitivity to the variability of the Indian Ocean sea surface temperature that varies annually, the ITCZ alludes to a relation with Fsos (Karmalkar et al., 2012).

**Figure C1:**
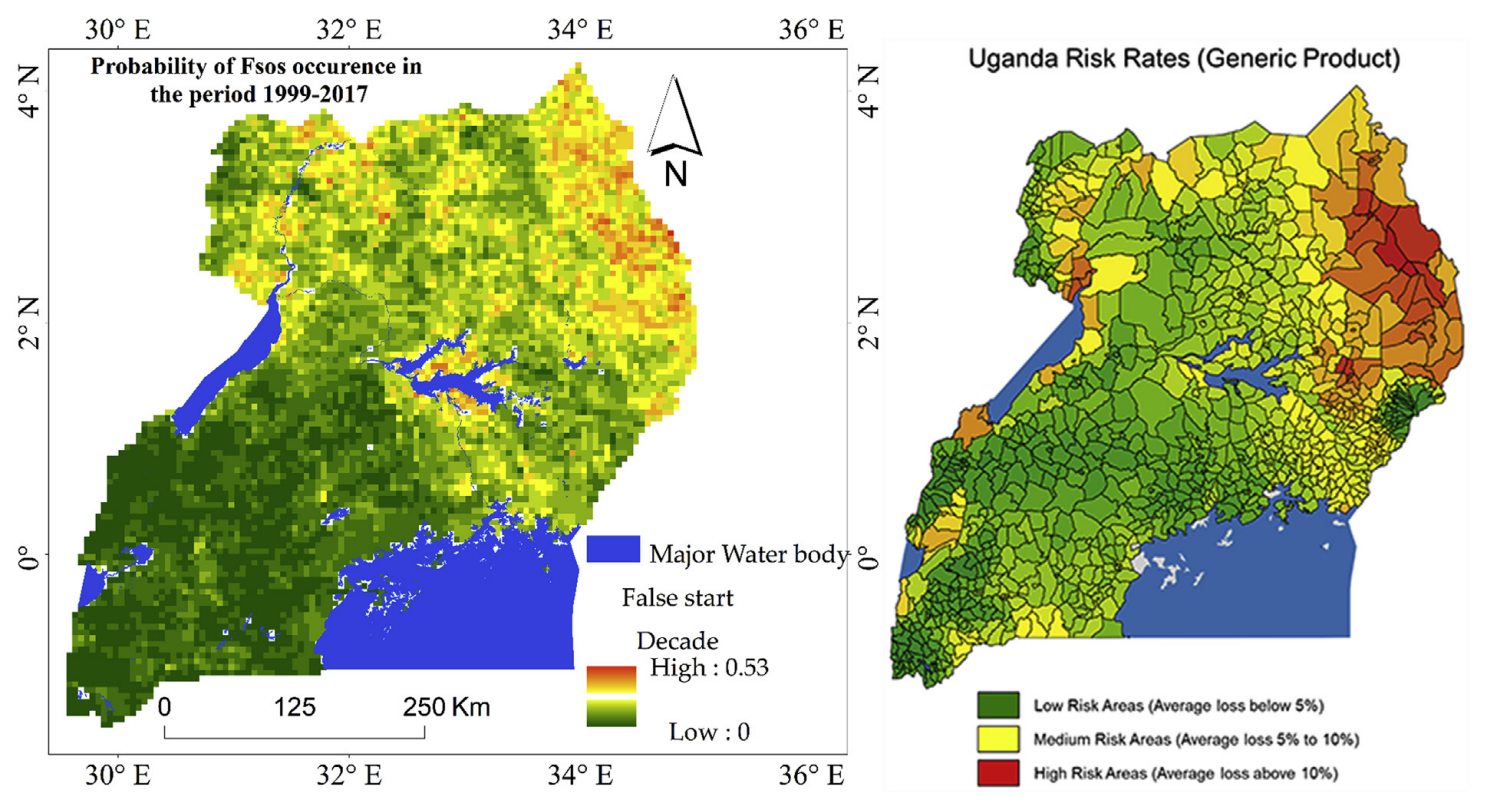
false start occurrence probability map (result from our study) and generic drought risk map during the overall growing season for Uganda as developed under the SUM-Africa project supported by the G4AW program of Netherlands Space Office (NSO).

Our finding that the areas identified to be at high risk of dry spell had high probability of Fsos is consistent with the result from the analysis by the Netherlands Space Organization (Netherlands Space Office, 2009), that mapped out areas prone to the occurrence of dry spell for the growing seasons for Uganda (Figure C1). The southern western exhibited low risk characterized by low probability of Fsos.

Our results complemented the finding by Orindi and Eriksen (2005) that the SoS for northern Uganda is highly variable and uncertain. The Fsos occurrence is therefore conceivably a component of the variability being experienced, thus creating conflict with the known farmers cropping calendar and affecting yields (Bryant et al., 2016). Subsequently impacting the socio-economic wellbeing of the farmers. Comparatively, in Nigeria, the timing of Fsos was shown to be on dates before the long term mean onset as observed in the SoS trigger dates (Benoit, 1977).

## 4 Conclusions

This study analysed the component of agronomic drought occurring during the onset of growing season in Uganda by characterizing areas that are prone to dry spell, providing insights on dry spell occurrence during the onset of the season with possibility of it being related to false start. The results showed that the probability of dry spell occurring were higher during the onset of the MAM than that of the SON season. The north eastern part of the country was at risk throughout the growing season over the period 1999–2017. These findings demonstrated the strength of the statistical parameters in mapping areas at risk of dry spell during the onset of the growing season providing a framework for the detection of the occurrence of false start in the first planting season in Uganda. Additionally, the characterization of the dry spell risk in relation to MAM and SON, presents valuable information for planning of rainfall dependent crop farming activities. Accordingly, the findings from the study are also beneficial in the design of agricultural insurance scheme supporting risk assessment, adaptation strategies and formulation of policies. False start tended to be associated with 7-9^th^ dekads with areas in northern and north eastern Uganda having higher probability than for the southern region.

The implication of these findings is that farmers have to be cautious and not to rush into planting immediately after onset of rainfall. It is advisable for farmers to monitor the onset of rainfall for at least ten days before concluding it as the start of the growing season. Therefore, this is a vital information for Uganda farmers in deciding the types of crops to grow and planting dates. Accordingly, the results clearly highlight the smallholder farmers’ needs to shift to planting drought tolerant crops varieties and integrate soil water management technologies.

Despite our results, it is important to investigate alternative methods that can be applied to detect false start of growing season. For instance, the inclusion of temperature and soil moisture parameters into such investigative analysis would enable net water balance to be taken into account. In the future, research on false start should further be motivated by prospects of assessing the duration of a dry spell after the false start date detection and interview of lager number (or at least 50) farmers per pixel to allow for evaluation of the severity of the impact. Furthermore, it is worth noting that even if the Maximum Value Composite (MVC) was applied in this study, many newer compositing methods use band quality flags more than max NDVI. Max NDVI can select off nadir pixels because of more of a side look than straight down (nadir). It is recommended that the influence of the choice of compositing methods on the outcome of analyses related to false start of the growing season be considered in future research.

## Declarations

### Author contribution statement

Emmanuel Ocen: Conceived and designed the experiments; Performed the experiments; Analyzed and interpreted the data; Wrote the paper.

de Bie C.A.J.M (Kees): Conceived and designed the experiments; Performed the experiments; Analyzed and interpreted the data; Contributed reagents, materials, analysis tools or data; Wrote the paper.

Charles Onyutha: Contributed reagents, materials, analysis tools or data; Wrote the paper.

### Funding statement

This work was supported by Nuffic NFP Scholarship programs.

### Data availability statement

Data included in article/supplementary material/referenced in article.

### Declaration of interests statement

The authors declare no conflict of interest.

### Additional information

No additional information is available for this paper.

## Figures and Tables

**Figure A 1:**
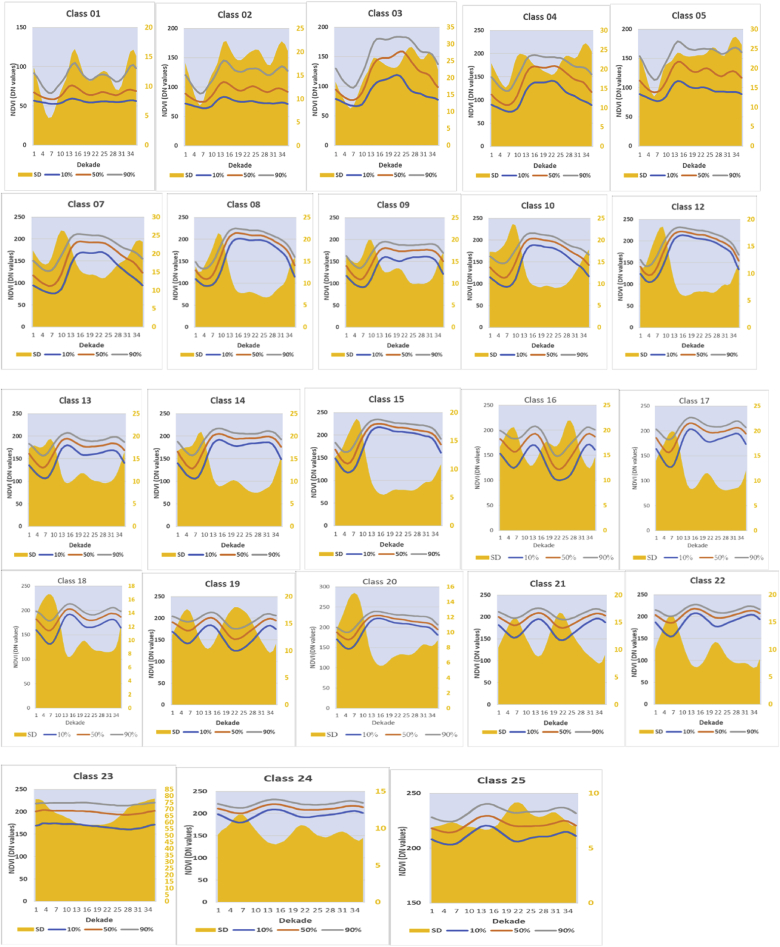
Temporal profile pattern for each of the classes generated from the unsupervised classification, describing the 10^th^, 50^th^, 90^th^ percentiles and standard deviation as indicator of variability during the growing season in Uganda from the year 1999 to 2017.

## Appendix

**Appendix A**: Extended legend for the unsupervised classified map (Figure 4).

**Appendix B**: Field interview data, collected to have farmers insight on the occurance of false start, perception on rainfall variability and allowing for its utility in definition of false start date through the annotation of the data into satellite data.

**Appendix C**: Visual comparison of false start probability map and risk of a dry spell during the overall growing season for Uganda conducted by Netherland Space Organization. When we compare our probability map and risk map developed under the NSO program, we realized that as it is in our finding the south western part of Uganda is less at risk of dry spell when compared to the north eastern part. These two studies used different dataset, but the spatial representation of risk displays similar characteristics across the country as shown in Figure C1 .
